# Plasmapheresis: Is it a potential alternative treatment for chronic urticaria?

**DOI:** 10.1515/med-2021-0399

**Published:** 2021-12-23

**Authors:** Laima Aleksandraviciute, Laura Malinauskiene, Kestutis Cerniauskas, Anzelika Chomiciene

**Affiliations:** Department of Chest Diseases, Immunology and Allergology, Institute of Clinical Medicine, Faculty of Medicine, Vilnius University, Santariskiu 2, Vilnius LT-08661, Vilnius, Lithuania; Department of Chest Diseases, Immunology and Allergology, Institute of Clinical Medicine, Faculty of Medicine, Vilnius University, Vilnius, Lithuania

**Keywords:** chronic urticaria, plasmapheresis, omalizumab, antihistamines, urticaria treatment

## Abstract

**Background:**

Chronic urticaria is a common disease. Plasmapheresis is an alternative treatment that can be appropriate for patients who are resistant to treatment with 2nd generation antihistamines or for whom treatment with omalizumab is unsuitable.

**Objective:**

To investigate the effect of plasmapheresis treatment in chronic urticaria.

**Methods:**

A retrospective analysis was performed based on the data of 98 patients suffering from refractory chronic urticaria who received plasmapheresis as an alternative treatment in Vilnius University’s Hospital Santaros Clinics from 2000 to 2020. The efficiency of the treatment was evaluated by clinical judgment.

**Results:**

58.2% of the patients exhibited a complete or significant response; of these, 37.8% had temporary relief of symptoms and 20.4% achieved disease remission; 41.8% showed no response to the plasmapheresis. Men (34.8%) had a tendency to achieve disease remission more often than women (16%) (*p* < 0.05). One patient did not finish the plasmapheresis treatment due to the symptoms’ exacerbation and treatment with omalizumab was initiated.

**Conclusion:**

Plasmapheresis is a safe and effective alternative treatment when traditional treatment is unavailable or does not relieve symptoms completely. Our data showed that plasmapheresis was effective in more than half of our patients.

## 1 Introduction

Chronic spontaneous urticaria (CSU) defined as the presence of wheals, angioedema, or both, which lasts for at least 6 weeks, affects patients’ quality of life due to recurrent symptoms. Recent studies have shown that CSU prevalence depends on the region and varies between 0.1–1.4% of the general population [1].

Although the main event in CSU is activation and degranulation of dermal mast cells, the pathogenesis of CSU remains mostly unclear. In recent years, more evidence shows that CSU can be an autoimmune disease as up to 40% of the patients have detectable histamine-releasing immunoglobulin G (IgG) autoantibodies directed against immunoglobulin E (IgE) or the high-affinity IgE receptor on mast cells and basophils or IgE autoantibodies to common structures (e.g., thyroid gland) [2].

International guidelines by European Academy of Allergology and Clinical Immunology (EAACI), the EU-founded network of excellence, the Global Allergy and Asthma European Network (GA^2^LEN), the European Dermatology Forum (EDF), and the World Allergy Organization (WAO) recommend oral non-sedating antihistamines, the dosage of which can be increased up to four times as a first-line treatment option for CSU. As around half of the CSU patients cannot be controlled on this treatment, biologic omalizumab (anti-IgE) or cyclosporine A are recommended, mainly because the efficacy of these drugs is demonstrated in randomized controlled trials. About 30% of the patients remain symptomatic at licensed doses of omalizumab 150 and 300 mg, even after a treatment period of over 6 months [3]. Patients treated with cyclosporine A achieve remission from 54 to 73% depending on the prescribed dose and treatment duration [4]. Various other treatments have also been used and described for CSU, but are not included in the current international guidelines.

As there is increasing evidence on the possible pathogenetic role for circulating autoantibodies in CSU, we aimed at evaluating the effectiveness of plasmapheresis and to find biomarkers of the effectiveness in our cohort of CSU patients.

## 2 Methods

In this retrospective study, we analyzed data of 98 CSU patients (75.5% women and 24.5% men) (Table 1) treated between August 2000 and March 2020. The mean age of CSU beginning was 39.8 years. CSU was diagnosed according to EAACI/GA^2^LEN/EDF/WAO guidelines for the definition, classification, diagnosis, and management of urticaria. All patients suffered from severe refractory to antihistamines CSU, lasting more than 6 weeks, and were treated with 4 times higher dose of 2nd generation antihistamines. Patients with inducible urticaria were excluded from the study. Before receiving plasmapheresis, ten patients were also treated unsuccessfully with omalizumab (available in Lithuania since 2016) and two patients with cyclosporine.

**Table 1:** Characteristics of 98 CSU patients treated with plasmapheresis

	Patients (*n* = 98)
Female	75 (76.5%)
Male	23 (23.5%)
Median age of CSU manifestation	39.8 years (±15.7)
Sensitivity to NSAIDs by history	17 (17.3%)
Food influence to CSU	7 (7.1%)
Other conditions	
Autoimmune thyroiditis	24 (24.5%)
Urticarial vasculitis	9 (9.2%)
Diabetes	6 (6.1%)
Oncological disease	6 (6.1%)
Atopic dermatitis	3 (3.1%)
IgA deficiency	2 (2.0%)

NSAID, non-steroidal anti-inflammatory drugs; CSU, chronic spontaneous urticaria.

One course of plasmapheresis consisted of six procedures which usually were performed every second day. During each procedure, 200–350 mL of plasma was removed and replaced with 750 mL of 0.9% normal saline within 2 h. The volume of removed plasma depended on the patients’ weight, height, and results of laboratory tests. Before procedure, patients underwent a complete blood test, total protein or albumin, creatinine, sodium, potassium, and calcium analyses. During one course of plasmapheresis, approximately 2,000 mL of plasma was removed.

Effectiveness evaluation of the plasmapheresis treatment was based on a modified global evaluation of treatment effectiveness [5]. Physician graded patients depending on how effective treatment had been in controlling the patients’ CSU. Patients were grouped into three groups (Table 2).

**Table 2:** Patient groups depending on clinical effect

Group	Clinical effect
Group 1	Remission: patients had no symptoms for at least 1 month, did not need antihistamines
Group 2	Temporary symptoms relief: patients had no symptoms for less than a month or used lower doses of antihistamines than usual
Group 3	No significant effect: the symptoms remained the same, used the same doses of antihistamines

The autologous serum skin test (ASST) was performed on 47 patients. ASST was performed according to EAACI/GA^2^LEN guidelines [6].

Anti-TPO antibodies and thyroid-stimulating hormone (TSH) tests were performed on 53 patients. The normal anti-TPO level was considered to be <5.61 kU/L. Normal TSH level was considered to be 0.4–4.0 mU/L.

The statistical program SPSS 20 was used for statistical analysis. Results are presented as mean values and standard deviations. Findings were compared between the groups using the Chi-square test as appropriate. *p* values < 0.05 were considered statistically significant.


**Ethical approval:** The study was approved by the Regional Ethics Review Board in Vilnius, Lithuania, and was conducted following ethical standards specified in the Declaration of Helsinki.

## 3 Results

**Figure 1:**
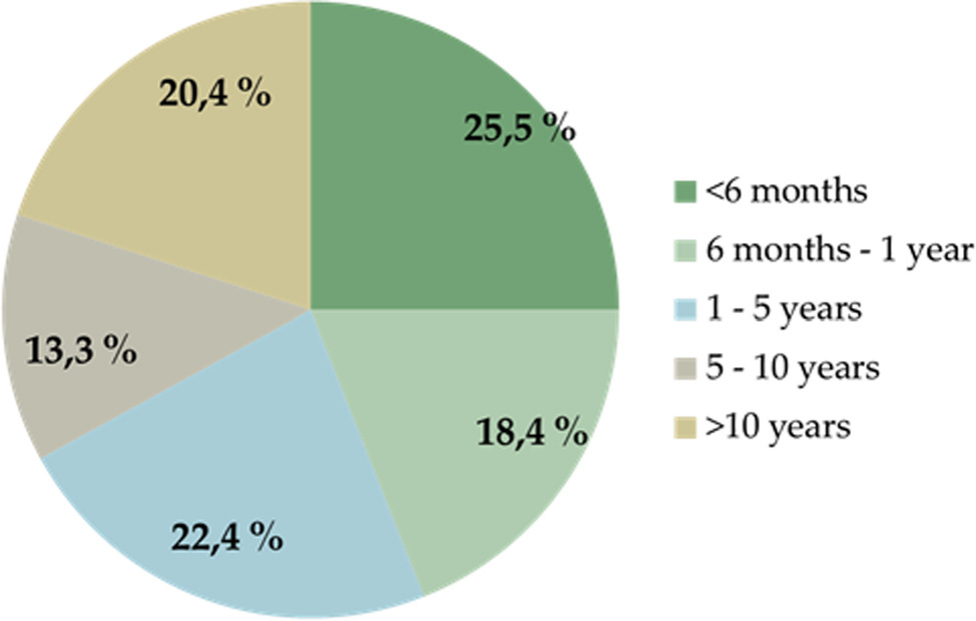
Duration of chronic spontaneous urticaria before plasmapheresis.

**Figure 2:**
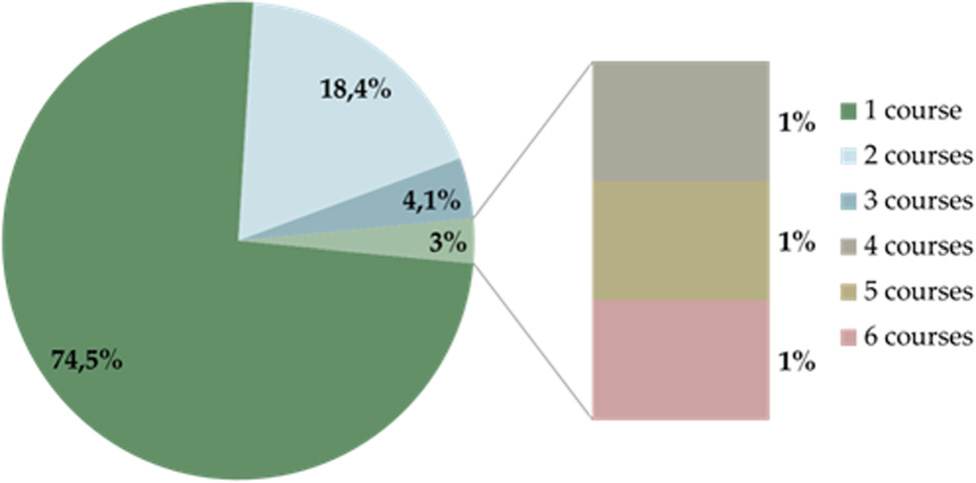
Number of plasmapheresis courses.

Plasmapheresis was initiated within 6 months of CSU symptoms appearing in 25.5% (25/98) of the patients (Figure 1). Most of the patients (74.5%), received 1 plasmapheresis course, 1 patient received 6 courses of plasmapheresis within 5 years (Figure 2). We repeated plasmapheresis only in case it was successful and patients were willing to perform it again.

The ASST was performed on 47 patients, 22 of them were positive (Table 3). We did not find any statistically significant differences in the plasmapheresis effectiveness in the group of patients with positive ASST (*p* = 0.065).

**Table 3:** Plasmapheresis effect in positive and negative ASST groups

Number of patients	Clinical effect
Positive autologous serum skin test group	
9 (40.9%)	Remission
9 (40.9%)	Temporary symptoms’ relief
4 (18.2%)	No effect
Negative autologous serum skin test group	
3 (12.0%)	Remission
13 (52.0%)	Temporary symptoms’ relief
9 (36.0%)	No effect

More than half of the patients were investigated for thyroid dysfunction. Anti-TPO antibodies and TSH tests were performed on 53 patients (Table 4). In a group of patients with increased anti-TPO antibodies, there was a tendency to achieve better disease control results than in patients with normal anti-TPO antibodies level. In a group of patients with increased anti-TPO antibodies, 25% achieved remission and 45.8% felt temporary symptoms’ relief after plasmapheresis. In those with normal anti-TPO antibodies, 24.1% achieved remission and only 27.6% felt temporary symptoms’ relief. However, the results were not statistically significant (*p* = 0.296).

**Table 4:** Thyroid dysfunction and plasmapheresis effect

Number of patients	Thyroid stimulating hormone level
36 (67.9%)	Normal
16 (30.2%)	Hypothyroidism
1 (1.9%)	Hyperthyroidism

Number of patients	Clinical effect
Patient with increased anti-TPO antibodies	
6 (25.0%)	Remission
11 (45.8%)	Temporary symptoms’ relief
7 (29.2%)	No effect
Patient with normal anti-TPO antibodies	
7 (24.1%)	Remission
8 (27.6%)	Temporary symptoms’ relief
14 (48.3%)	No effect

**Figure 3:**
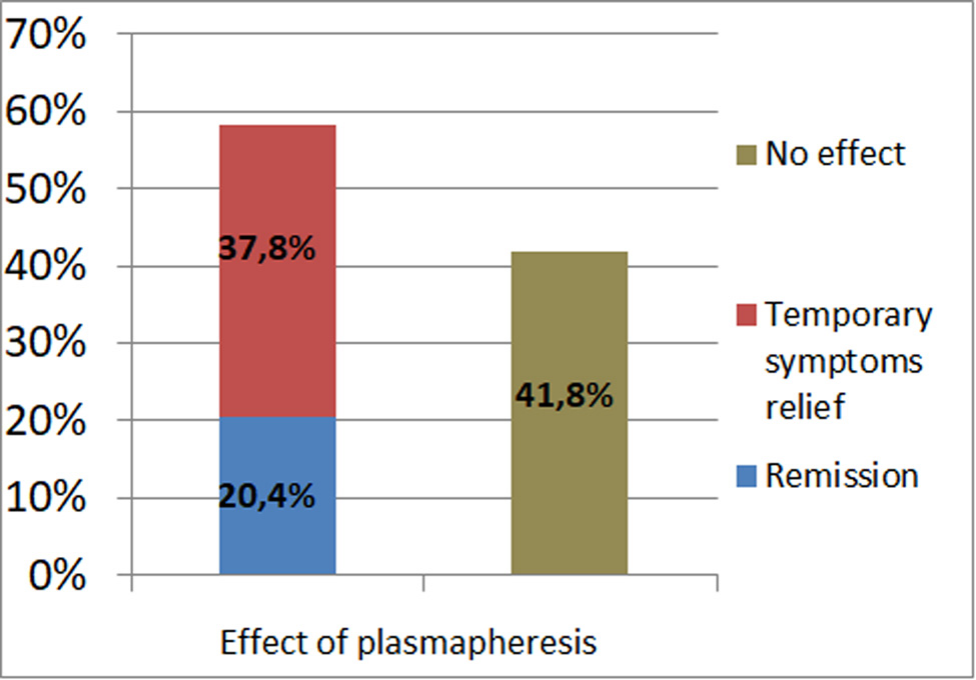
Effect of plasmapheresis.

58.2% of the patients exhibited a complete or significant response; of these, 37.8% had temporary symptoms’ relief and 20.4% achieved disease remission; 41.8% showed no response to plasmapheresis (Figure 3). The study showed that men (34.8%) tended to achieve disease remission more often than women (16%) (*p* < 0.05).

## 4 Discussion

In most cases, CSU is treated based on EAACI/GA^2^LEN/EDF/WAO recommended algorithm. Although, in clinical practice a bunch of different alternative treatment options are used. One of them is plasmapheresis.

Plasmapheresis can be performed by centrifugation or filtration [7]. During filtration plasmapheresis, whole blood passes through a filter and plasma components are separated from the larger cellular components of red blood cells, white blood cells, and platelets. Double–filtration plasmapheresis removes medium to large molecules, including various antibodies [8]. Centrifugation is usually performed by blood bankers. Although in our clinic, centrifugal plasmapheresis was performed on patients with CSU. Only a few case reports with positive outcomes of the plasmapheresis treatment in severe CSU have been published. Patients with CSU often have circulating IgG autoantibodies against high-affinity IgE receptors or IgE. Recently there is some evidence of IgE antibodies against self-antigens (e.g., TPO) [9]. It is presumed that CSU is caused by autoantibodies activating mast cells. We can expect the plasmapheresis treatment to be effective because through this treatment medium and large molecular substances such as IgG and IgE are removed from circulation.

If there is a suspicion of autoimmune or autoreactive urticaria, a screening test such as ASST can be performed. Basophil activation assays and basophil mast cell histamine release tests are used to detect functional autoantibodies against IgE and FcεRIα [10]. Antibody specificity is confirmed by immunoassay. In clinical practice, laboratory tests are not performed routinely [11]. Our study results showed that the plasmapheresis treatment was more effective in patients with positive ASST than in those with negative ASST, but the data were not statistically significant (*p* = 0.065). Some studies have also shown that reduction of functional auto-antibodies via plasmapheresis had a temporary benefit in some, severely affected patients [12,13].

There is little information about the effect of plasmapheresis in patients with CSU and thyroid dysfunction. A case report of successful resistant CSU treatment with plasmapheresis in a patient who also had thyroid gland disorder was published in Poland [13]. We found that in a group of patients with increased anti-TPO antibodies, there was a tendency to achieve better treatment results (*p* = 0.296). Even though plasmapheresis seems to be more effective for patients with increased anti-TPO antibodies, further investigation needs to be done.

CSU may develop in children and adults, but it mostly affects adults between the age of 20 and 40 years [14]. In our study, the average age of disease manifestation was 39.8 years (±15.7). Since the disease affects young, working-age people, and has a negative impact on life quality, it is very important to start effective treatment as soon as possible. Based on our data there was no correlation between the duration of urticaria and the plasmapheresis effect (Table 5).

**Table 5:** Plasmapheresis effect depending on duration of CSU

Clinical effect	Duration of CSU				
	<6 months	6 months–1 year	1–5 years	5–10 years	>10 years
Remission	3 (12%)	2 (11.1%)	5 (22.7%)	4 (30.8%)	6 (30.0%)
Temporary symptoms’ relief	9 (36.0%)	9 (50.0%)	9 (40.9%)	3 (23.1%)	7 (35.0%)
No effect	13 (52.0%)	7 (38.9%)	8 (36.4%)	6 (46.1%)	7 (35.0%)

Omalizumab is available in Lithuania as a treatment for CSU since 2016, that is why we used plasmapheresis quite often in antihistamines-resistant CSU. Nowadays, CSU patients are treated with plasmapheresis only when omalizumab is ineffective or the patient refuses to be treated with it. Patients for whom plasmapheresis was effective were willing to repeat it when CSU reoccurred. One patient received even six courses of plasmapheresis. The initial four courses of plasmapheresis were successful, but after the fifth ineffective course, she was treated with omalizumab, which was partially effective. The patient underwent a sixth course of plasmapheresis but it did not have a significant effect. Since then, the patient is being treated with omalizumab. The patient’s symptoms are less severe but full remission has not been reached.

Omalizumab is a third-line add-on therapy to second-generation H1-antihistamines. It is an effective and well-tolerated treatment option for CSU patients who remain symptomatic despite second-generation H1-antihistamines treatment. In our study, ten patients received unsuccessful treatment with omalizumab before plasmapheresis. For half of them, plasmapheresis was effective; 4 felt temporary symptoms’ relief and 1 achieved disease remission after 4 years of CSU. Some patients who received plasmapheresis initially did not need further treatment with omalizumab. 25 of our analyzed patients did not receive omalizumab after plasmapheresis. For the majority of these 25 patients, plasmapheresis was effective; 12 felt the relief of symptoms, and 9 achieved disease remission (Table 6).

**Table 6:** Plasmapheresis effect in group of patients for whom omalizumab was ineffective

Clinical effect	Duration of CSU				
	<6 months	6 months–1year	1–5 years	5–10 years	>10 years
Remission	2 (28.6%)	2 (40.0%)	2 (40.0%)	1 (50.0%)	2 (33.3%)
Temporary symptoms’ relief	5 (71.4%)	1 (20.0%)	2 (40.0%)	1 (50.0%)	3 (50.0%)
No effect	0 (0.0%)	2 (40.0%)	1 (20.0%)	0 (0.0%)	1 (16.7%)

Plasmapheresis is an alternative treatment that is effective in some cases, as shown by available data [8,12,13,15]. Grattan et al. published an analysis on eight patients with severe, unremitting, CSU who underwent plasmapheresis. Urticarial activity score was evaluated by wheal numbers, duration, frequency, and itchiness (each symptom scored from 0 to 4, the maximum total score being 16). Six of eight patients had a positive response to the treatment [12]. In our study, a large group of patients were analyzed. 58.2% of all patients exhibited a complete or significant response after one course of plasmapheresis, of which 37.8% had temporary symptoms relief and 20.4% achieved disease remission. 41.8% showed no response to plasmapheresis.

Plasmapheresis is a safe treatment option. The procedure is contraindicated for patients who do not tolerate central line placement, are hemodynamically unstable, or have hypocalcemia. Major possible adverse events of plasmapheresis are hypothermia, numbness, hypotension, and puncture site hematoma. In rare cases arrhythmia, convulsions, or loss of consciousness can occur. There were no severe adverse events in our analyzed group of patients. Only one patient discontinued plasmapheresis due to the exacerbation of the symptoms.

There are numerous gaps in the knowledge about the pathogenesis of CSU. Large, controlled, well-designed, and randomized trials have not been conducted for most of the alternative agents including plasmapheresis. Despite very effective biological treatment, a rational, patient-based approach can still be used that is based on evidence, the potential for adverse effects, costs, and patient preferences.

Our study has some limitations. The retrospective single-center analysis was performed without objective evaluation criteria (e.g., Urticaria Activity Score summed over 7 days, Dermatology Life Quality Index, and disease-specific Chronic Urticaria Quality of Life Questionnaire).

## 5 Conclusion

In our study, more than half of all patients exhibited a complete or significant response to the treatment. Based on our data, plasmapheresis is a safe, effective alternative treatment. Although there was a tendency of better results in patients with positive ASST and increased anti-TPO antibodies, more studies are needed to evaluate the effect of plasmapheresis for patients with autoimmune thyroid disease and autoreactive urticaria.
